# Supplementary material for: Complementarity of PALM and SOFI for super-resolution live-cell imaging of focal adhesions
Source: Nat Commun. 2016 Dec 19;7:13693. doi: 10.1038/ncomms13693 (PMC5187410; doi:10.1038/ncomms13693)
Supplement: Supplementary Information — Supplementary Figures 1-16, Supplementary Notes 1-5 and Supplementary References 1-13. [file ncomms13693-s2.pdf]

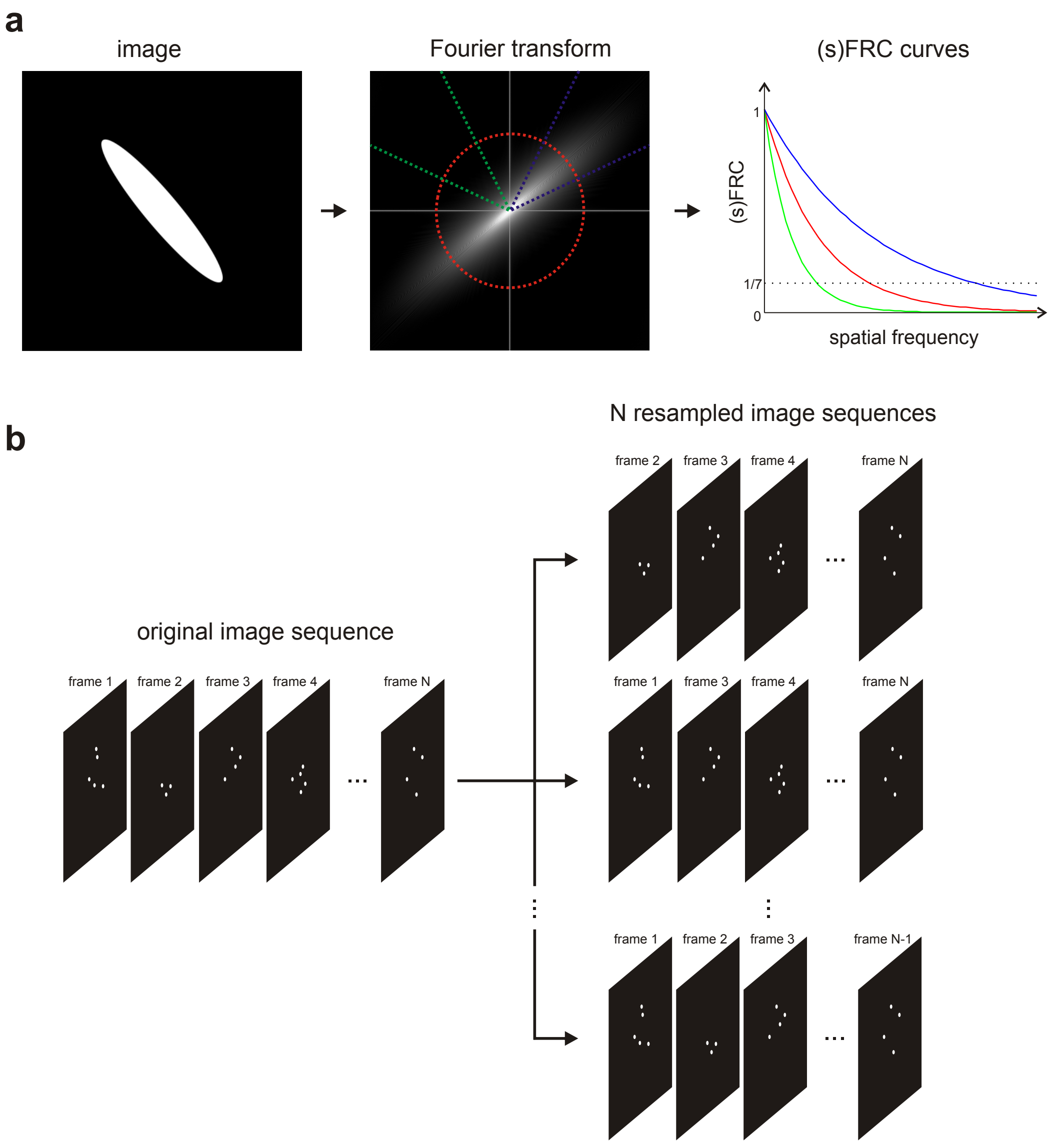

Supplementary Figure 1: Resolution and SNR metrics. (a) Illustration of the sectorial Fourier ring correlation to obtain a measure of the resolution. (b) Illustration of the delete-1 jackknife resampling method to obtain a measure for the signal-to-noise ratio (SNR).

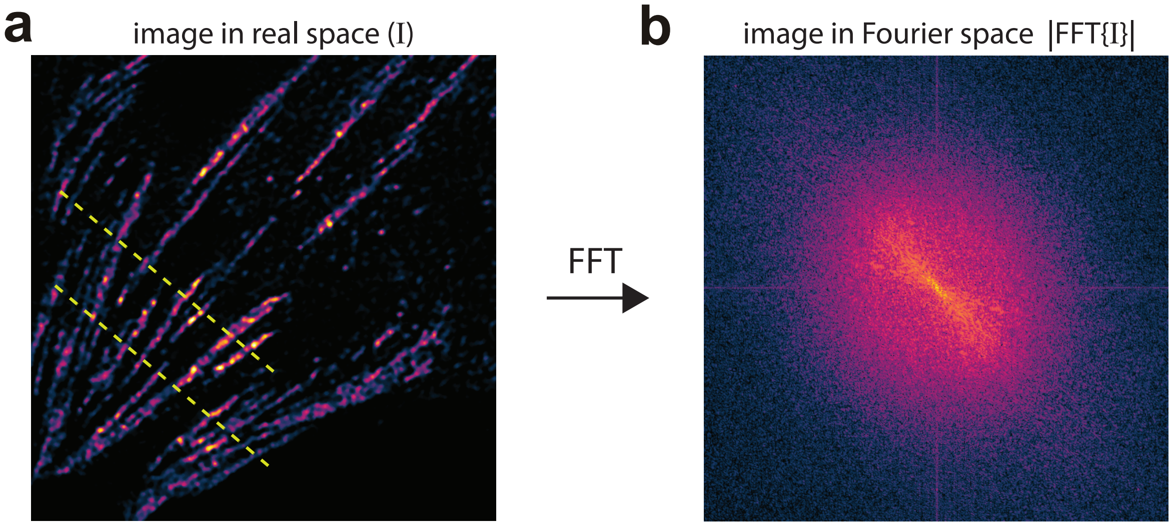

Supplementary Figure 2: Anisotropic frequency content. (a) 4th order bSOFI image of a fixed MEF cell expressing paxillin labeled with psCFP2. The high spatial frequency changes of intensity appear mostly in one direction (marked by the yellow line). (b) Most of the high frequency content in Fourier space appears along the same direction. The color map "morgenstemning" was applied [1].

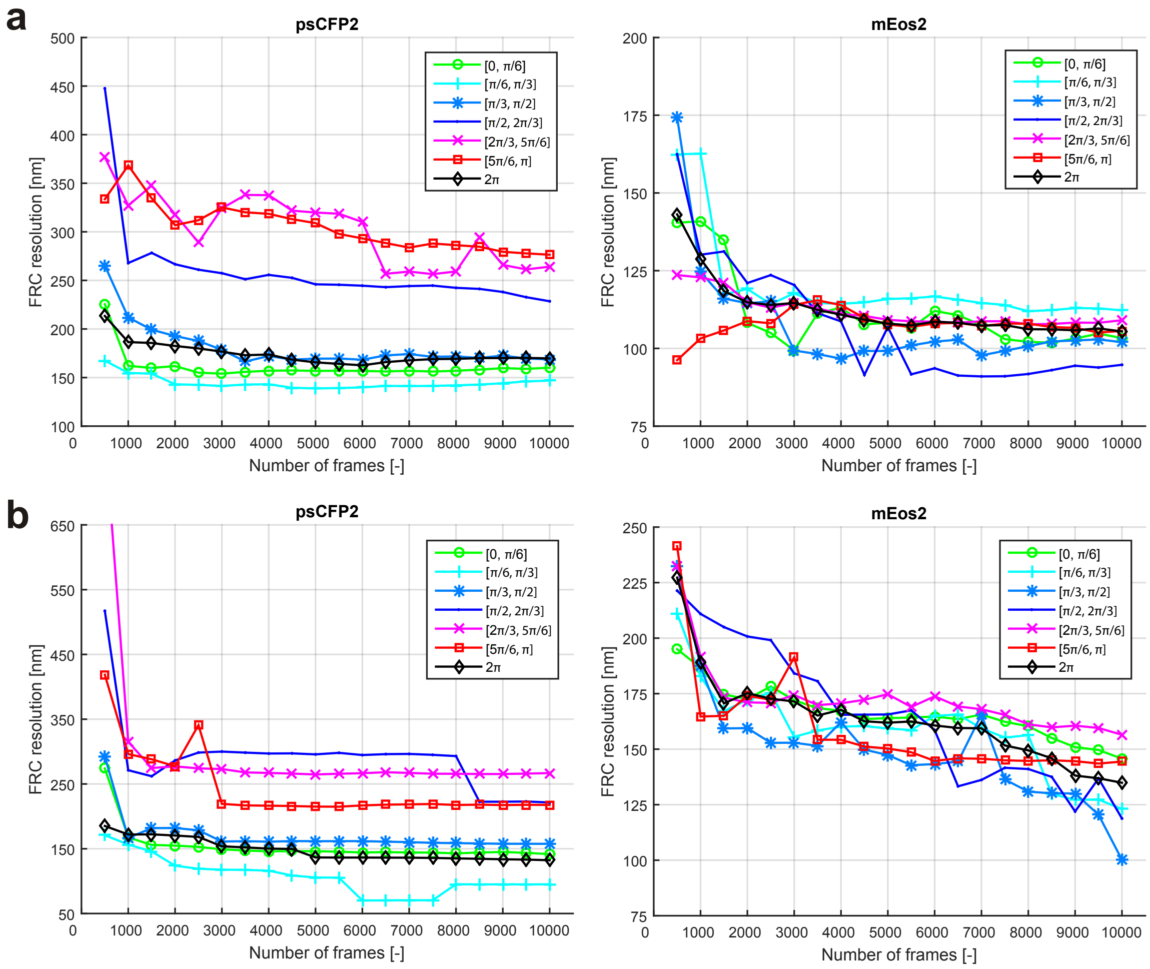

Supplementary Figure 3: The sFRC as a function of the number of frames for the data shown in Figure 1. (a) The sFRC for 6 different sectors, together with the FRC, obtained from PALM. (b) The sFRC for 6 different sectors, together with the FRC, obtained from SOFI.

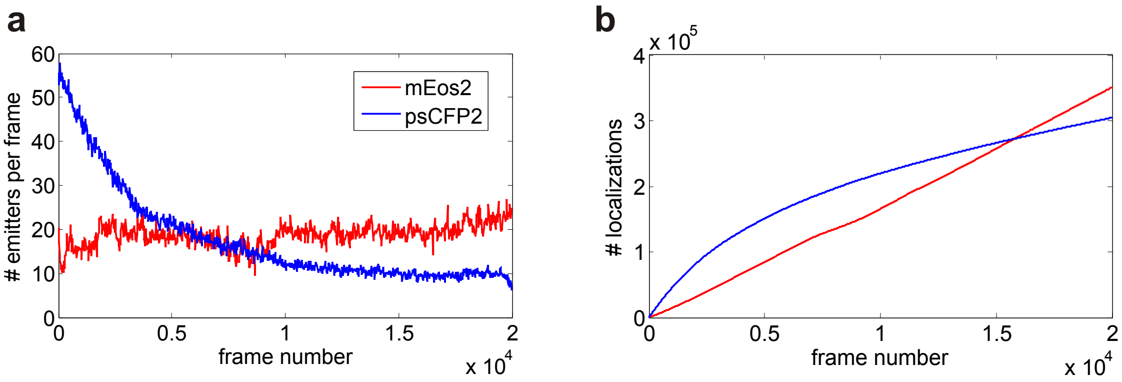

Supplementary Figure 4: Time evolution of the number of fluorophores in the data shown in Figure 1. (a) The number of detected fluorophores per frame (averaged over 20 frames) as a function of the frame number. (b) The number of localizations as a function of the number of frames. Note that a fluorophore can be detected in several consecutive frames, giving rise to a single localization.

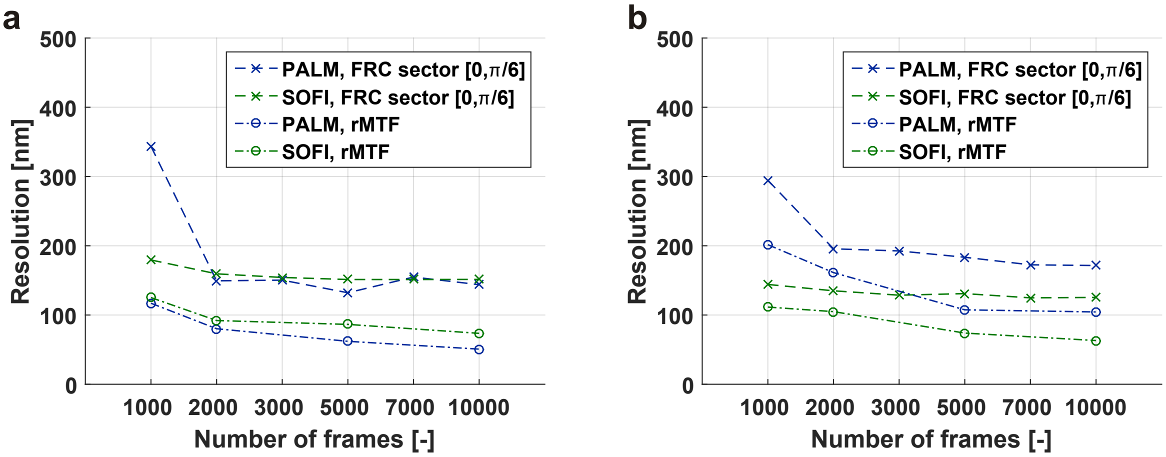

Supplementary Figure 5: sFRC and resolution based on MTF both measured on the same simulated dataset. From all the simulated data sets shown in Figure 2, this Supplementary Figure corresponds to the dataset with $I_{\rm on} = 100$ photons, and 20000 frames. (a) density 800 molecules/μm^2, (b) density 1200 molecules/μm^2.

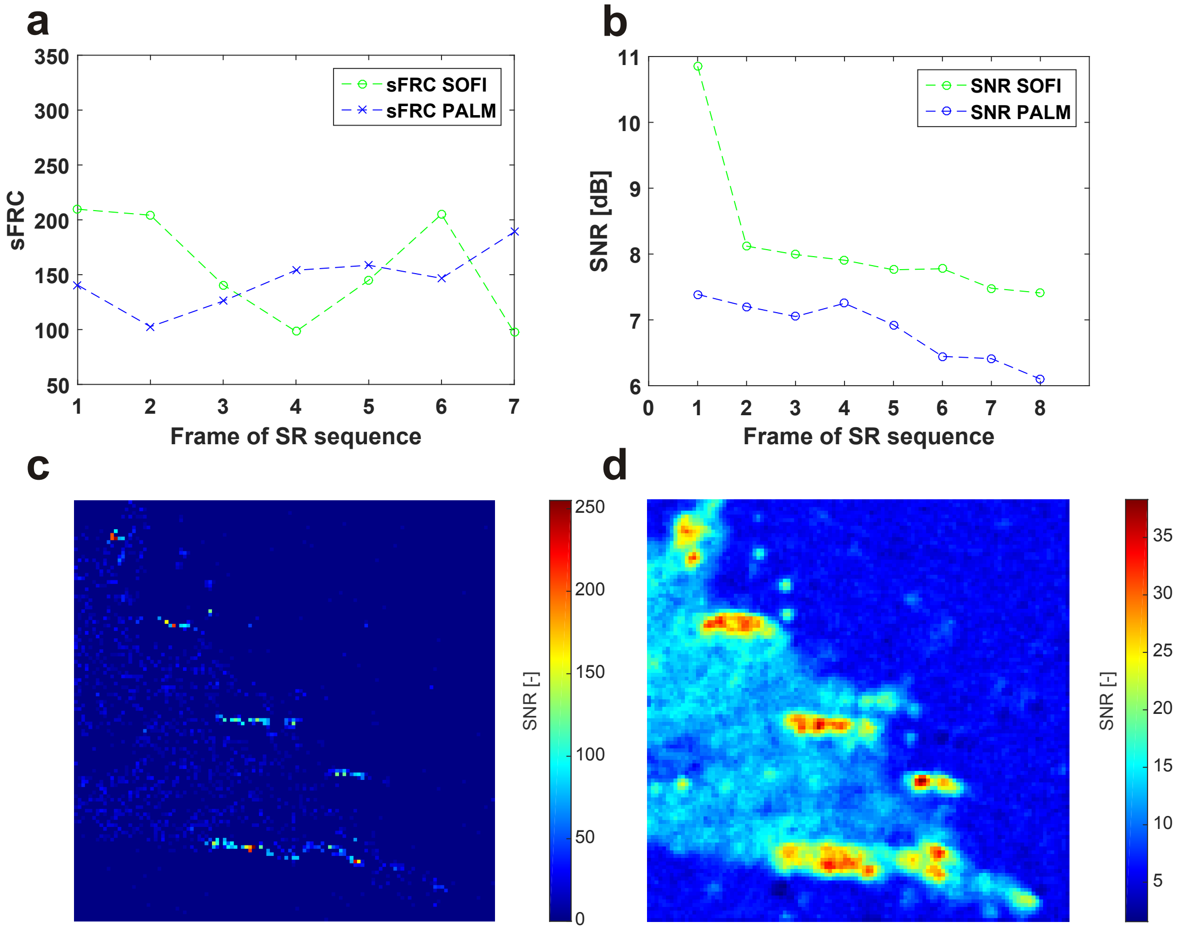

Supplementary Figure 6: SNR and sFRC calculated on SOFI and PALM movie of a living MEF cell expressing paxillin labeled with mEos2. Each image is reconstructed from 1000 camera frames with 10 ms exposure time, resulting in a 10 s temporal resolution. (a) sFRC values for each super-resolved SOFI/PALM frame. (b) average SNR for each super-resolved SOFI/PALM frame. (c) PALM (d) SOFI SNR map of the first frame of the PALM/SOFI output sequence.

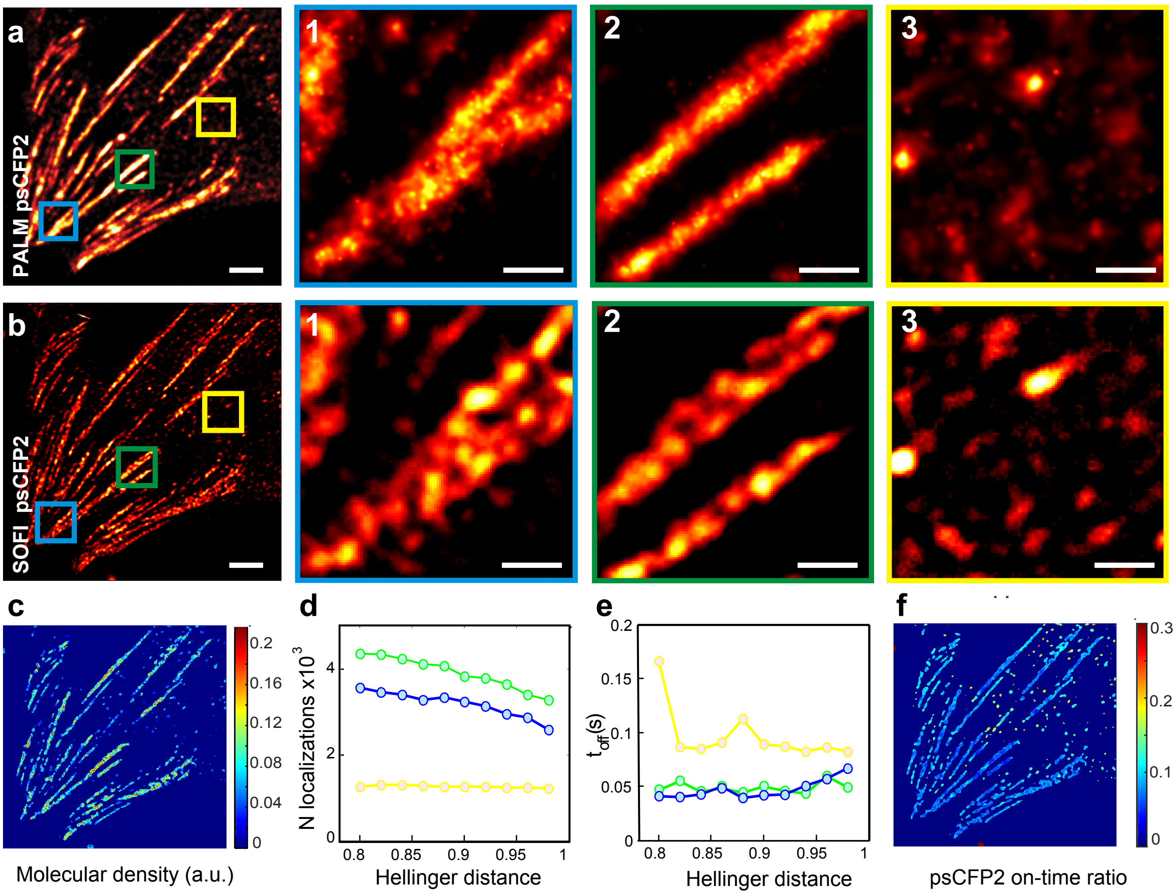

Supplementary Figure 7: Quantitative imaging with PALM and SOFI. (a-b) PALM and SOFI images of a fixed MEF cell expressing paxillin labeled with psCFP2. Panels 1-3 are corresponding zoom-ins for the PALM or SOFI images. (c,f) SOFI analysis yields a fluorophore density and on-time ratio map as described in Supplementary Note 2.3. (d,e) Blinking events in PALM data can be detected by merging localizations that are sufficiently close in space and time. This analysis yields the blink corrected number of localizations N and the corresponding average off-time t_{off} between blinks, shown as a function of the distance threshold for merging localizations. The PALM images were rendered as probability maps (see Methods). Scale bars: (a,b) 2 μm , (1-3) 0.5 μm.

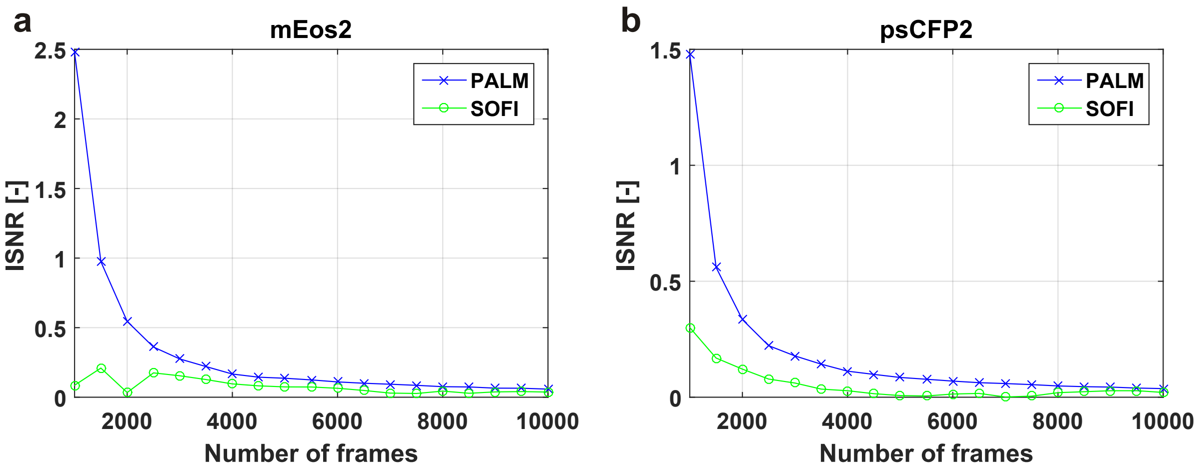

Supplementary Figure 8: SNR convergence rate measured on PALM and SOFI images of a fixed MEF cell expressing paxillin labeled with (a) mEos2 and (b) psCFP2.

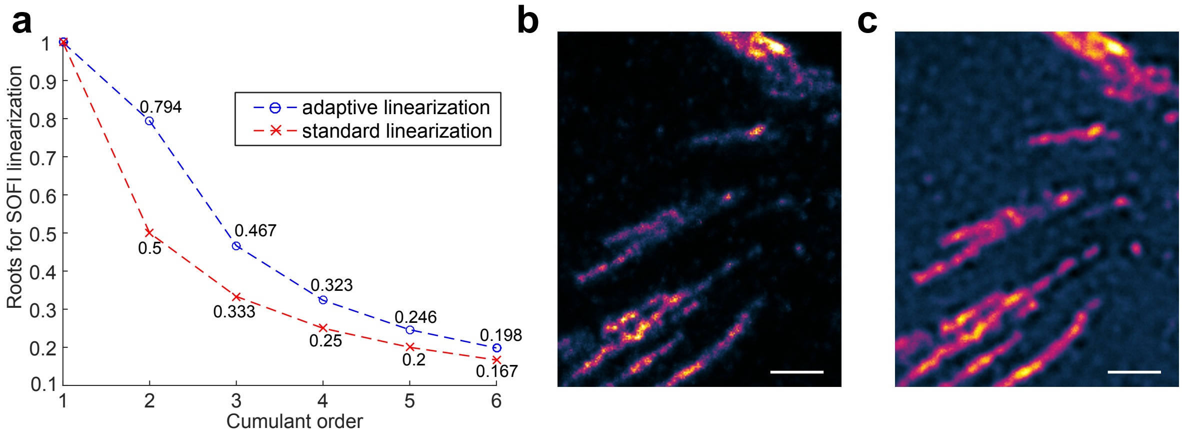

Supplementary Figure 9: Enhanced bSOFI. Images of fixed MEF cells expressing paxillin labeled with mEos2 obtained from a raw image sequence of 20,000 frames. (a) Roots for SOFI standard and adaptive linearization. (b) 4th order bSOFI using a novel linearization (sFRC=134 nm). (c) 4th order bSOFI using standard linearization (sFRC=166 nm). Dynamic range is reduced too much which leads to lower SNR and deconvolution artifacts in the low SNR background regions. Scale bars: 1 μm

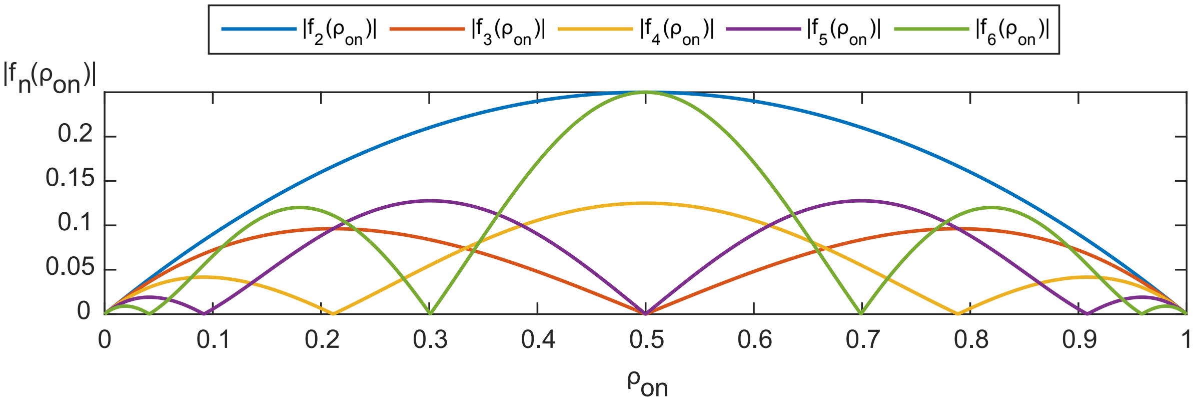

Supplementary Figure 10: On-time ratio polynomial of 2nd to 6th order as a function of the on-time ratio.

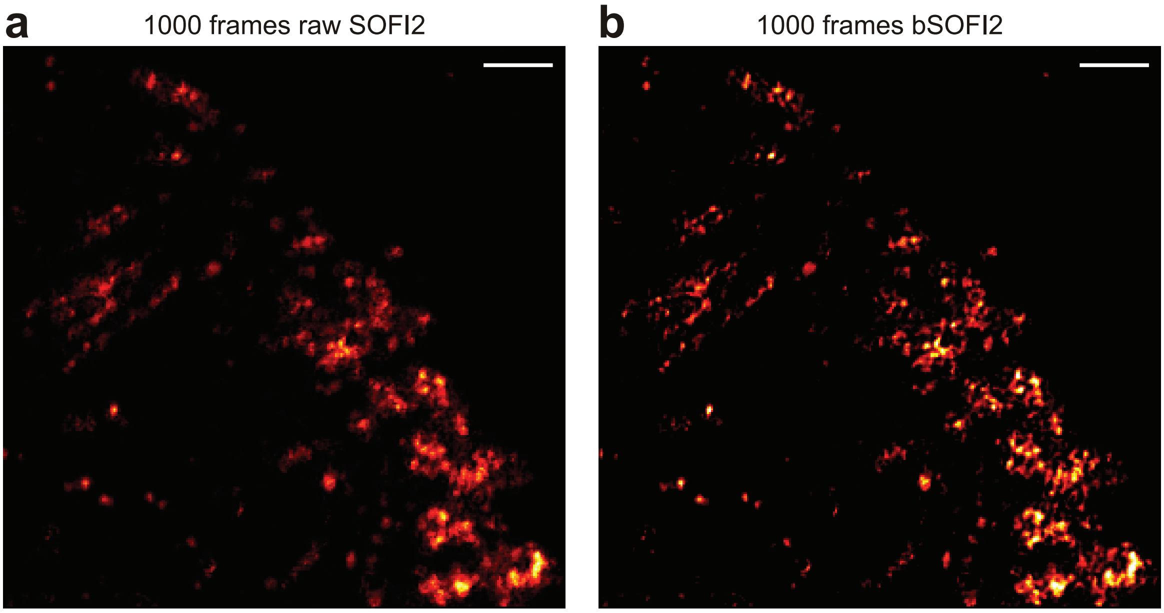

Supplementary Figure 11: Deconvolving SOFI images. (a) Raw SOFI image (2nd order) reconstructed from the first 1000 frames of the input image sequence. (b) bSOFI image, i.e. raw SOFI image in (a) deconvolved and linearized using the procedure described in Supplementary Note 2.2. Scale bars: 2 μm

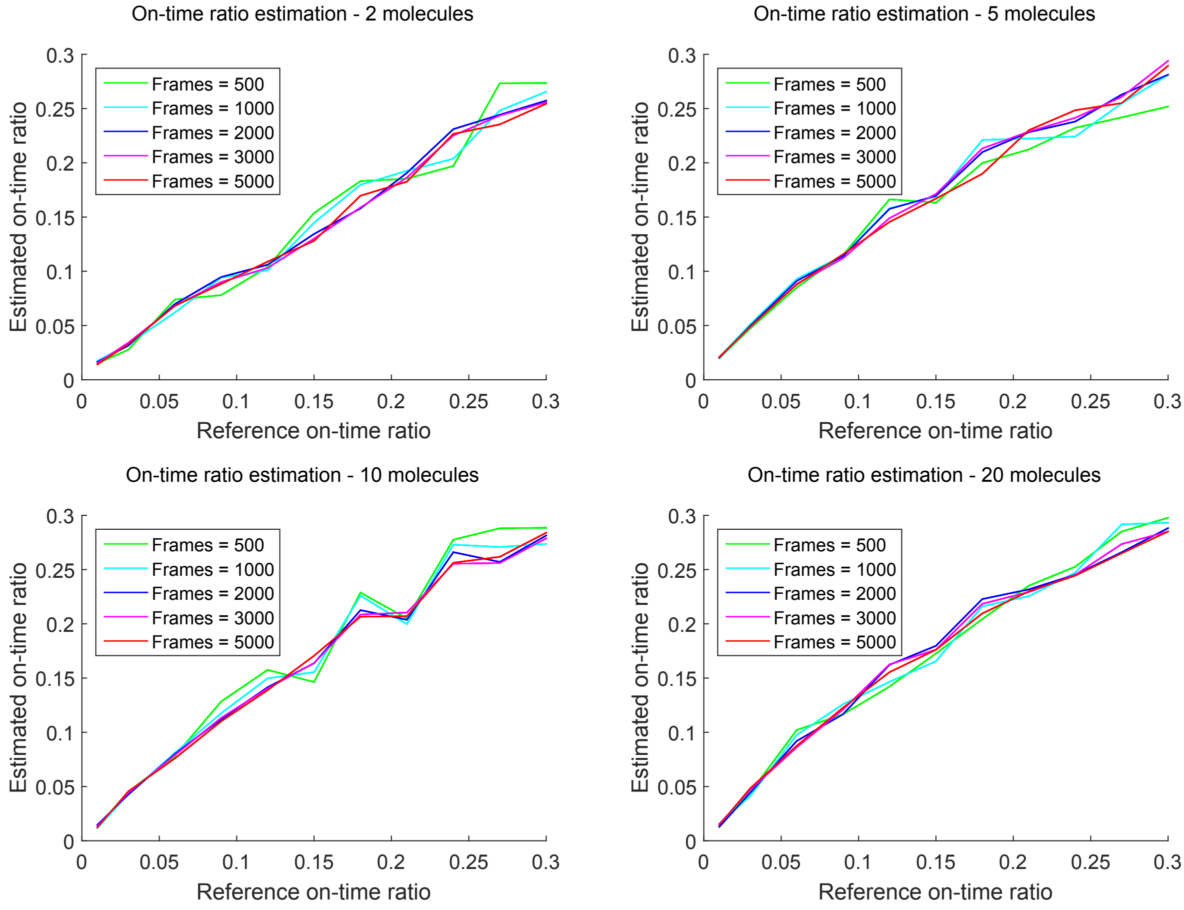

Supplementary Figure 12: On-time ratio estimation. Precision of the on-time ratio estimation was tested using simulated image stacks with a varying number of frames.

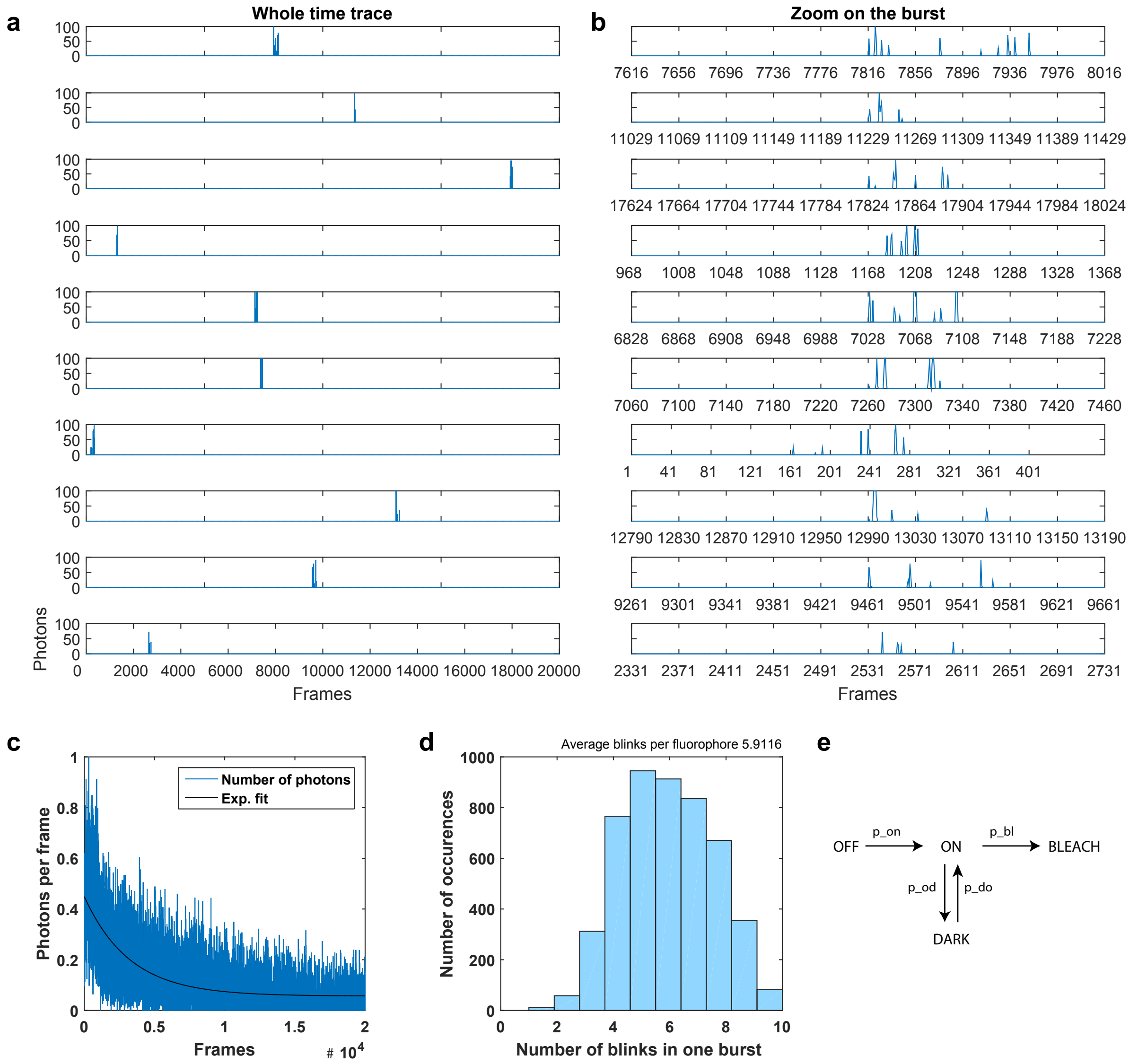

Supplementary Figure 13: Simulation of photophysics of fluorophores. (a) Time traces of the first 10 fluorophores are shown. Each fluorophore goes randomly into the on-state and quickly blinks several times, i.e. switches between an on-state (bright) and a dark state. (b) The zoom shows these blinking events in detail. The frequency and duration of these blinks is modeled according to measurements of mEos2 photokinetics in [2]. (c) Number of photons as a function of frames. (d) Number of blinking events in on "burst". During the "burst" each fluorophore blinks several times. (e) A schematic drawing of the four state photophysics model.

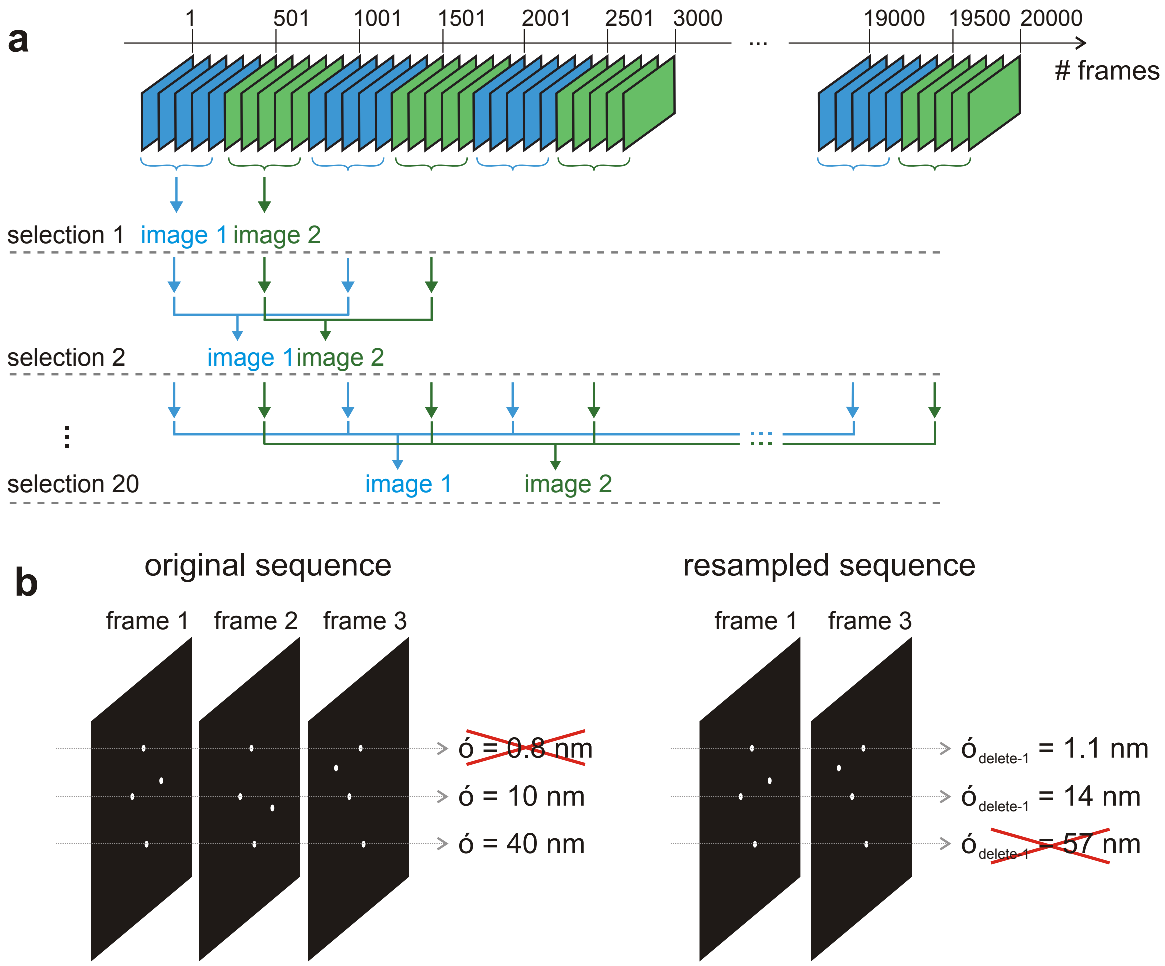

Supplementary Figure 14: Details of the implementation of resolution and SNR metrics. (a) Illustration of the frame selection procedure for the sFRC calculation. (b) Illustration of the effect on the localization precision of leaving out a frame in the SNR calculation.

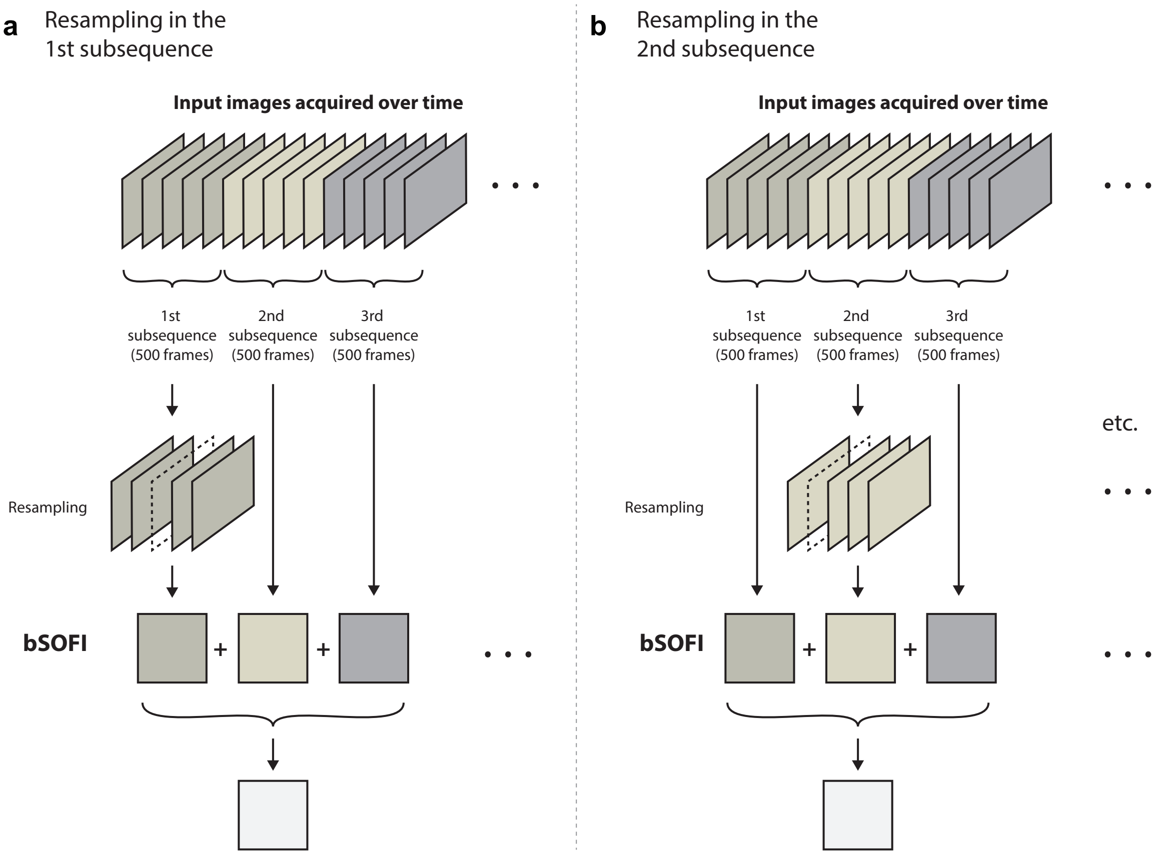

Supplementary Figure 15: Estimating SNR using jackknife resampling. (a) The input image sequence is divided into subsequences. In the first step, the resampling is performed within the first subsequence. Each time one frame of the first subsequence is left out, the bSOFI image is calculated and summed up with the bSOFI images calculated from the remaining subsequences. (b) When all the resampling possibilities are evaluated in the first subsequence, the algorithm starts resampling the second subsequence.

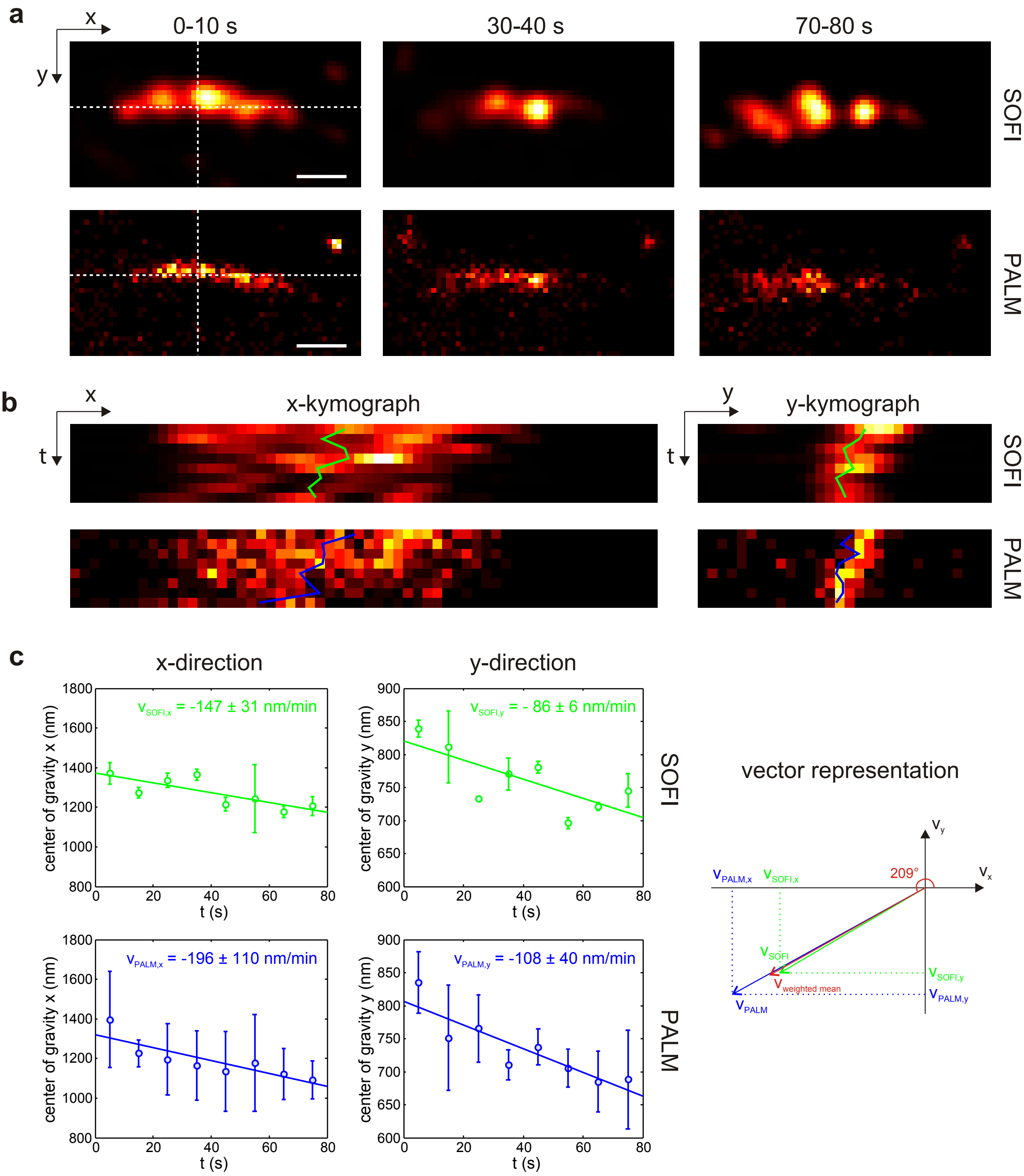

Supplementary Figure 16: Live cell imaging with PALM and bSOFI. (a) PALM and SOFI image of living MEF expressing paxillin labeled with mEos2. (b) Region of interest indicated in (a) at different time points. Each image is reconstructed from 1000 camera frames with 10 ms exposure time, resulting in a 10 s temporal resolution. (c) Kymographs along the lines indicated in (b). (d) Focal adhesion edge position as a function of time determined from the kymographs in (c). The edges were identified as the first pixel from both sides in the kymograph with a value that exceeds half of the maximum value. The velocities have been obtained by a linear fit. Scale bars: 0.5 μm

Supplementary Note 1: Resolution estimation using sectorial Fourier ring correlation

Estimating the resolution in single molecule localization microscopy (SMLM) is challenging, since it depends on several parameters such as the labelling density, the localization precision, and the sample structure. An interesting resolution metric for SMLM is the Fourier ring correlation (FRC) [3, 4]. However, the FRC implicitely assumes that the sample structure is isotropic, while focal adhesions are known for their typical pattern giving rise to anisotropic Fourier spectra (Supplementary Figure 1,2). We have therefore adapted the FRC metric and introduced sectorial Fourier ring correlation (sFRC) to account for the effect of specific focal adhesion patterns.

1.1 From FRC to sFRC

To calculate the FRC, a SMLM dataset is first split into two stochastically independent subsets for generating two SMLM images $I_1(x, y)$ and $I_2(x, y)$. Next, the Fourier transforms $\hat{I}_1(q, \phi)$ and $\hat{I}_2(q, \phi)$ of these two images are calculated (with polar coordinates in frequency space given by magnitude q and phase ϕ). The FRC is then calculated as

$$FRC(q) = \frac{\sum_\phi \hat{I}_1(q,\phi)\hat{I}_2(q,\phi)^*}{\sqrt{\sum_\phi |\hat{I}_1(q,\phi)|^2 \sum_\phi |\hat{I}_2(q,\phi)|^2}} \tag{1}$$

correlating $\hat{I}_1(q, \phi)\hat{I}_2(q, \phi)^*$ over a full circular path at a constant magnitude q. For low spatial frequencies, the FRC is close to 1, whereas for high spatial frequencies, the FRC decays to 0. Finally, after applying a smoothing step, the FRC resolution can be calculated as the inverse of the radial frequency for which the curve drops below 1/7 (i.e. the radial cut-off frequency), as suggested in [4].

Evaluating the cross-correlation of $\hat{I}_1(q, \phi)$ and $\hat{I}_2(q, \phi)$ along a circular path entails an insensitivity to pronounced directional variations in the spatial frequency content, as shown in Supplementary Figure 1. This occurs especially for our images containing specific patterns of cell adhesions, which have strongly directional imbalanced Fourier spectra (see Supplementary Figure 1a). We therefore introduced the sFRC as a generalization of the FRC. As already suggested in [4], with this generalized metric, the correlation taken over a full circle is replaced by the correlation over a sector with an angular extend of $\Delta\phi$:

$$sFRC(q, \Delta\phi) = \frac{\sum_{\Delta\phi} \hat{I}_1(q,\phi)\hat{I}_2^*(q,\phi)}{\sqrt{\sum_{\Delta\phi} |\hat{I}_1(q,\phi)|^2 \sum_{\Delta\phi} |\hat{I}_2(q,\phi)|^2}} \tag{2}$$

This sFRC concept takes into account the major anisotropy of the image spectrum (see Supplementary Figure 1, 2). Obviously, the classical FRC metric is easily recovered by calculating the sFRC for a sector with an angular extend of 2π. As a guideline, we suggest to evaluate the sFRC in 12 sectors with an angular extend of $\pi/12$, compromising between an improvement in sampling of the direction and a decrease in the amount of data.

1.2 Selecting two stochastically independent data subsets

The (s)FRC calculation requires the SMLM data to be split in two stochastically independent subsets, in order to render two stochastically independent SMLM images. First, the full image sequence that consists of N frames is split into K subsequences (containing N/K frames) with K an even number.

Next, K subsequences are split into two subsets of these K subsequences (as shown in Supplementary Figure 14). Each of the two subsets is used to generate one super-resolved

image for the (s)FRC calculation. The selection of subsequences into 2 subsets should be done carefully. In case of a random selection, the second subset may contain almost no SMLM data due to photobleaching. This problem can be largely avoided by selection of K subsequences in an alternating way, i.e. creating two subsets of odd and even image subsequences (Supplementary Figure 14).

1.3 sFRC applied to SOFI

Although the FRC metric has been conceived for SMLM, we also applied the (s)FRC metric to super-resolution optical fluctuation (SOFI) images. In order to minimize the effect of photobleaching, a bleaching correction was applied to the input image sequence. The average fluorescence per frame was calculated and fitted by an exponential fit. Images of the input image sequence are then weighted by coefficients given as an inverse of the exponential fit [5]. SOFI processing takes into account temporal relations between consecutive frames, therefore a random division of the input image sequence into two subsets is not possible. We used the alternative approach described above. After splitting the full image sequence into K subsequences (Supplementary Figure 14a), a SOFI image is calculated for each subsequence separately. After selecting a first set of $K/2$ subsequences, a SOFI image is obtained by summing the SOFI images corresponding to these subsequences. Applying the same procedure to the second $K/2$ subsequences yields two SOFI images for the (s)FRC calculation. As the SOFI analysis requires consecutive frames, subsequences with a sufficient number of frames should be chosen. We have chosen subsequences of 500 frames each.

Supplementary Note 2: Enhanced bSOFI and on-time ratio estimation

Cell adhesion and its dynamics have been assessed for the first time with SOFI up to the 6th order. Besides a gain in resolution we also addressed the impact on contrast. Using bSOFI up to the sixt order translates into a decrease in contrast necessitating to readdress this question to fully exploit the potential of bSOFI imaging.

We modified the bSOFI algorithm [6] by introducing a linearization for a better compensation of the intrinsic nonlinearity of SOFI. Additionally, this takes into account the on-time ratio and linearizes the response to detected intensity. In summary, we show that this step improves the attainable resolution assessed by the sFRC as well as the contrast (Supplementary Figure 9).

2.1 SOFI theory

SOFI is based on higher order statistics, i.e. spatial-temporal cross-cumulants calculated from a time series of images of stochastically blinking emitters. The resolution improvement is given by the properties of these cumulants and described in a seminal paper by J. Enderlein and coworkers [7].

Assuming N independently fluctuating emitters, the detected intensity can be described as

$$I(\mathbf{r},t) = \sum_{k=1}^{N} \epsilon_k U(\mathbf{r}-\mathbf{r}_k) s_k(t) + b(\mathbf{r}) + n(\mathbf{r},t) \tag{3}$$

where ϵ_k is the molecular brightness, $U(\mathbf{r}-\mathbf{r}_k)$ is the PSF of the optical system, $s_k(t)$ denotes a switching function (normalized fluctuation sequence, $s_k(t) \in \{0,1\}$), $b(\mathbf{r})$ is a constant background, and $n(\mathbf{r},t)$ represents an additive noise. The sample is assumed to be stationary during the image acquisition. Generally, spatial-temporal cumulants can be calculated with various time lags. For reducing the computational complexity and ensuring the maximum of the signal, we used zero time lags. As shown in [8], virtual pixels can be calculated in between the physical pixels acquired by the camera using cross cumulants and followed by a flattening operation (i.e. assigning proper weights to virtual pixels) [8, 9, 10]. Using the properties of cumulants, the n-th order cumulant with zero time lag applied to Eq. 3 can be written as

$$\kappa_n\{I(\mathbf{r},t)\} = \sum_{k=1}^{N} \epsilon_k^n U^n(\mathbf{r}-\mathbf{r}_k)\kappa_n\{s_k(t)\} + \kappa_n\{b(\mathbf{r})\} + \kappa_n\{n(\mathbf{r},t)\} \tag{4}$$

For $(n \geq 2)$, under the assumption of uncorrelated noise and stationary background, the terms $\kappa\{b(\mathbf{r})\}$ and $\kappa\{n((r),t)\}$ will cancel out.

In the photoactivated localization microscopy (PALM) photo-physical model, the emitter activation is assumed as non-reversible, however, since the emitter is activated, it exhibits several quick blinking events prior to be finally bleached [2]. On a shorter time scale (within one subsequence of input dataset), the emitter fluctuates. If the emitter fluctuates between two different states (an on-state S_{on} and a dark state S_{off}), we can define the on-time ratio as

$$\rho = \frac{\tau_{\text{on}}}{\tau_{\text{on}} + \tau_{\text{off}}} \tag{5}$$

where τ_{on} and τ_{off} are the characteristic lifetimes of S_{on} and S_{off} states. The n-th order cumulant $\kappa_n\{s_k(t)\}$ is in this model described by a Bernoulli distribution with probability ρ_{on} [6] and approximated by an n-th order polynomial function of the on-time ratio (further referred to as a cumulant function)

$$f_n(\rho_{\text{on}}) = \rho_{\text{on}}(1-\rho_{\text{on}})\frac{\partial f_{n-1}}{\partial \rho_{\text{on}}} \tag{6}$$

Under these conditions, the n-th order spatial-temporal cross-cumulant can be approximated as

$$\kappa_n\{I(\mathbf{r},\mathbf{t})\} \approx \epsilon^n f_n(\rho_{\text{on}}) \sum_{k=1}^{N} U^n(\mathbf{r}-\mathbf{r}_k) \tag{7}$$

2.2 Linearization and higher order SOFI

The molecular brightness as described in Eq. 7 is raised to the n-th power. High order cumulant images exhibit fluorescent spots of high brightness which are masking less bright structural details. The non-linear response to molecular brightness limits the use of high order cumulants with consequences on resolution enhancement and contrast. S. Geissbuehler et al. [6] proposed balanced SOFI (bSOFI) which allows one to linearize the nonlinear brightness response. Firstly, the n-th order cumulant image is deconvolved. Secondly, the brightness response is linearized by taking the n-th root of the deconvolved cumulant image. This approach has proven efficient for 2D and 3D super-resolution imaging [6, 11].

When using SOFI up to the sixth order, we need to readdress the linearization by taking into account the contribution of $f_n(\rho_{\text{on}})$ in Eq. 7. Supplementary Figure 10 shows the cumulant function dependence on the on-time ratio ρ_{on} for different orders. In the case of a 4th order cumulant and $\rho_{\text{on}} = 0.2$, the cumulant function decreases. Under these conditions, the contrast of the 4th cumulant image is attenuated. The resulting image is flat and the dynamic range is reduced strongly which leads to a loss of signal-to-noise ratio (SNR). In general, the SNR drops with increasing orders limiting the maximum available resolution enhancement. To overcome this problem, we introduced a novel linearization procedure which takes into account the influence of the cumulant function and linearizes the response to the detected intensity.

The first four cumulants can be written as

$$\begin{aligned}
g_1 &\approx \epsilon(\mathbf{r}) f_1(\rho_{\text{on}}) \sum_{k=1}^{N} U(\mathbf{r}-\mathbf{r}_k) + \kappa_1\{b(\mathbf{r})\} + \kappa_1\{n(\mathbf{r},t)\} \\
g_2 &\approx \epsilon^2(\mathbf{r}) f_2(\rho_{\text{on}}) \sum_{k=1}^{N} U^2(\mathbf{r}-\mathbf{r}_k) \\
g_3 &\approx \epsilon^3(\mathbf{r}) f_3(\rho_{\text{on}}) \sum_{k=1}^{N} U^3(\mathbf{r}-\mathbf{r}_k) \\
g_4 &\approx \epsilon^4(\mathbf{r}) f_4(\rho_{\text{on}}) \sum_{k=1}^{N} U^4(\mathbf{r}-\mathbf{r}_k)
\end{aligned} \tag{8}$$

and the on-time ratio polynomials up to the sixth order are

$$f_1(\rho_{\text{on}}) = \rho_{\text{on}} \tag{9}$$
$$f_2(\rho_{\text{on}}) = \rho_{\text{on}}(1-\rho_{\text{on}}) \tag{10}$$
$$f_3(\rho_{\text{on}}) = \rho_{\text{on}}(1-\rho_{\text{on}})(1-2\rho_{\text{on}}) \tag{11}$$
$$f_4(\rho_{\text{on}}) = \rho_{\text{on}}(1-\rho_{\text{on}})(1-6\rho_{\text{on}}+6\rho_{\text{on}}^2) \tag{12}$$
$$f_5(\rho_{\text{on}}) = \rho_{\text{on}}(1-\rho_{\text{on}})(1-2\rho_{\text{on}})(12\rho_{\text{on}}^2-12\rho_{\text{on}}+1) \tag{13}$$
$$f_6(\rho_{\text{on}}) = \rho_{\text{on}}(1-\rho_{\text{on}})(120\rho_{\text{on}}^4-240\rho_{\text{on}}^3+150\rho_{\text{on}}^2-30\rho_{\text{on}}+1) \tag{14}$$

Once the on-time ratio is estimated (as described in the next section), the value of the on-time ratio polynomial for a given cumulant order is calculated by Eq. 10-14. In order to correct for the amplified brightness without compromising the resolution, the cumulants have to be deconvolved first, as shown in [6]. The correction factor for a deconvolved n-th order cumulant image $\hat{g}_n$ is $1/f_n(\rho_{\text{on}})$ and we can write

$$\frac{\hat{g}_n}{f_n(\rho_{\text{on}})} = \hat{g}_n^{\frac{log_{10}(\hat{g}_n/f_n(\rho_{\text{on}}))}{log_{10}(\hat{g}_n)}} \tag{15}$$

Instead of taking the n-th root, the corrected, adaptively linearized cumulant image $\bar{g}_n$ is

$$\bar{g}_n = \hat{g}_n^{\frac{1}{n}\frac{log_{10}(\hat{g}_n/f_n(\rho_{\text{on}}))}{log_{10}(\hat{g}_n)}} \tag{16}$$

The roots for linearization of cumulants up to 6th order (linearization curve) and the difference in the final bSOFI images are shown in Supplementary Figure 9. The red line in Supplementary Figure 9a represents the standard linearization where the n-th order cumulant is linearized by taking the n-th root [1]. The corrected roots for our novel linearization are shown in blue (Supplementary Figure 9a).

The bSOFI algorithm contains an inherent deconvolution step [6]. Raw SOFI images were deconvolved using the MATLAB function "deconvlucy" and linearized according to the linearization procedure described above. The number of iterations for the deconvolution was set to 10 (standard settings). The PSF was modeled by a 2D Gaussian function and the FWHM was estimated from the data using the procedure described in [6]. Supplementary Figure 11 shows a comparison of a raw SOFI image and a bSOFI image if 1000 frames are used for the reconstruction. For a low number of frames and low signals, the low SNR areas may exhibit some deconvolution artifacts, which is usually an indication that more frames are required for the reconstruction. More advanced deconvolution with regularization can be considered in the future for further improvement of the results.

2.3 On-time ratio estimation

Higher-order cumulants contain information about the photo-physics of the emitters. Combining SOFI images of different cumulant orders, molecular parameter maps can be extracted such as on-time ratio, molecular brightness, and molecular density [6], which we applied to assess the dynamics of cell adhesions. Geissbuehler et al. [6] used three cumulant images (2nd, 3rd, and 4th order) to estimate the on-time ratio. Here we present an estimation of the on-time ratio using only two cumulant images (2nd and 3rd order).

If we assume spatially varying but locally constant on-time ratios and molecular brightness, the cumulants can be approximated by [6]

$$g_1(\mathbf{r}) \approx \epsilon(\mathbf{r})f_1(\rho_{\text{on}})N(\mathbf{r})\mathcal{E}_V\{U(\mathbf{r})\} + \kappa_1\{b(\mathbf{r})\} + \kappa_1\{n(\mathbf{r},t)\} \tag{17}$$

$$g_2(\mathbf{r}) \approx \epsilon^2(\mathbf{r})f_2(\rho_{\text{on}})N(\mathbf{r})\mathcal{E}_V\{U^2(\mathbf{r})\} \tag{18}$$

$$g_3(\mathbf{r}) \approx \epsilon^3(\mathbf{r})f_3(\rho_{\text{on}})N(\mathbf{r})\mathcal{E}_V\{U^3(\mathbf{r})\} \tag{19}$$

$$g_4(\mathbf{r}) \approx \epsilon^4(\mathbf{r})f_4(\rho_{\text{on}})N(\mathbf{r})\mathcal{E}_V\{U^4(\mathbf{r})\} \tag{20}$$

where $\mathcal{E}_V\{U^n(\mathbf{r})\}$ is the expectation value of $U^n(\mathbf{r})$, $N(\mathbf{r})$ is the number of molecules inside a detection volume V centered at $\mathbf{r}$. The second (g_2) and third (g_3) order cumulant images can be related as

$$g_3 = \frac{\mathcal{E}_V\{U^3(\mathbf{r})\}}{\mathcal{E}_V\{U^2(\mathbf{r})\}^{3/2}}\frac{1}{N^{1/2}(\mathbf{r})}\frac{f_3(\rho_{\text{on}})}{f_2^{3/2}(\rho_{\text{on}})}g_2^{3/2} \tag{21}$$

Substituting Eq. 10 and Eq. 11 into Eq. 21 leads to

$$g_3 = K \frac{1 - 2\rho_{\text{on}}}{\sqrt{\rho_{\text{on}}(1 - \rho_{\text{on}})}} g_2^{3/2} \tag{22}$$

where $K = \frac{\mathcal{E}_V\{U^3(\mathbf{r})\}}{\mathcal{E}_V\{U^2(\mathbf{r})\}^{3/2}} \frac{1}{N^{1/2}(\mathbf{r})}$.
For the on-time ratio ρ_{on}, we obtain the solution

$$\rho_{\text{on}} = \frac{1}{2} \left(1 \pm \frac{\sqrt{4K^2 g_2^3 g_3^2 + g_3^4}}{4K^2 g_2^3 + g_3^2} \right) \tag{23}$$

As shown in Supplementary Figure 10, the on-time ratio polynomial is symmetric around $\rho_{\text{on}} = 0.5$, thus Eq. 23 has two possible solutions. To estimate ρ_{on}, we first determine the constant K. The number of molecules $N(\mathbf{r})$ can be estimated using the second order cumulant and the first order cumulant after background subtraction ($\tilde{g}_1$).

$$N(\mathbf{r}) = \frac{\mathcal{E}_V\{U^2(\mathbf{r})\}}{\mathcal{E}_V\{U^1(\mathbf{r})\}^2} \frac{(1 - \rho_{\text{on}})}{\rho_{\text{on}}} \frac{\tilde{g}_1}{g_2} \tag{24}$$

Approximating the imaging PSF by a 3D Gaussian profile, we can write [6]

$$\mathcal{E}_V\{U^n_{\text{3DGauss}}(\mathbf{r})\} = \frac{c(\sigma_{x,y}, \sigma_z)}{n^{3/2}} \tag{25}$$

where $c(\sigma_{x,y}, \sigma_z)$ is a constant depending on the spatial extend of the PSF. Analogously, approximating the PSF near the interface in a total internal reflection (TIR) configuration by a lateral 2D Gaussian profile and an axial exponential profile, we obtain

$$\mathcal{E}_V\{U^n_{\text{TIR}}(\mathbf{r})\} = \frac{c(\sigma_{x,y}, \sigma_z, d_z)}{n^2} \tag{26}$$

where d_z represents the penetration depth of the TIR illumination [6]. The outcome of this analysis has been implemented into our SOFI code inducing the expected contrast gain.

To show the accuracy of the above described on-time ratio estimation, we performed tests on simulated data. Supplementary Figure 12 shows the results with varying number of frames and 2, 5, 10, and 20 molecules randomly distributed within the PSF volume. The simulation for each reference on-time ratio was repeated 20 times. Each estimated on-time ratio is an average over these 20 calculations.

2.4 Molecular density estimation

The density and molecular on-time ratio maps in Figure 5 and Supplementary Figure 7 were calculated by taking Eq. 18, 19, 20 and solving this system of equations pixel-wise for $\epsilon(\mathbf{r})$, ρ_{on}, and $N(\mathbf{r})$ as described in [1]. Figure 5 and Supplementary Figure 7 show color-coded densities and molecular on-time ratios overlaid with the 4th order bSOFI image as a transparency mask. This pixel-wise estimation is not relevant for image regions which contain only background noise. Therefore, the linearized bSOFI image is used as a transparency mask to cancel out the background regions. The bSOFI image was linearized using our novel adaptive linearization procedure described above.

We performed simulations in order to evaluate the reliability of the SOFI based molecular parameter estimation and the PALM based molecule counting analysis (see Supplementary Note 5) shown in Figure 5g. Using simulations and the photo-physical model described in Methods and in Supplementary Note 4.2, we simulated a square of size 1x1μm randomly populated by

fluorescent molecules with a given molecular density in the range 50 - 1600 molecules per μm^2. We generated image sequences of these blinking fluorescent molecules of different length (1000, 2000, and 5000 frames) and performed SOFI molecular density estimation and PALM molecule counting analysis. Each combination of the simulated (ground truth) density and the length of the image sequence was repeatedly generated and analyzed 10 times. Figure 5g shows the average and the standard deviation calculated over these measurements. This demonstrates the advantage of our complementary approach for quantitative imaging. PALM based density estimation works well for low molecular density areas, regardless of the number of frames, while SOFI based density estimation outperforms PALM for high molecule densities and high frame numbers.

Supplementary Note 3: Signal-to-noise ratio estimation using statistical resampling

Imaging dynamics of cell adhesions trades spatial against temporal resolution with an impact on SNR. Therefore, we characterized the SNR in order to ensure a sufficient image quality. We estimated the SNR in our focal adhesion images by making use of a general approach based on statistical resampling applied to SOFI as well as PALM.

3.1 Delete-1 jackknife resampling

The SNR of SOFI images can be calculated by delete-1 jackknife resampling [12], i.e. N new datasets equal to the number of raw images N of the original dataset are generated, but each new dataset leaves out a single image in these sequences, as shown in Supplementary Figure 1b. Each new dataset is used to generate a new SOFI image, yielding N new SOFI images. For each pixel value $I(x,y)$ of the original SOFI image, N new $I_n(x,y)$ values are generated. The level of uncertainty associated to each pixel $I(x,y)$ can be quantified using the SNR per pixel, defined as

$$\mathrm{SNR}(x,y) = \frac{I(x,y)}{\sqrt{\mathrm{var}\{I(x,y)\}}} \tag{27}$$

The jackknife mean estimator is

$$\bar{I}(x,y) =< I_n(x,y) > \tag{28}$$

The jackknife variance estimator is

$$\mathrm{var}\{I(x,y)\} = (N-1) < (I_n(x,y) - \bar{I}(x,y))^2 > \tag{29}$$

3.2 SNR estimation on PALM data

Although originally introduced for SOFI, the SNR can also be determined for SMLM data, since SMLM images can be rendered in a pixelated fashion (e.g. as a 2D histogram). Moreover, the SMLM localization procedure does not need to be repeated N times. It might be sufficient to localize the molecules only once from the original dataset, and afterwards just rendering N SMLM images by removing the localizations that correspond to the frame that is "deleted". However, there is a caveat: the same emitter can appear during several consecutive frames. This means that deleting its localization when one of these frames is deleted, is not necessarily correct if one imposes an upper limit on the localization precision. The reason is that the localization precision could still be sufficiently small for the localization to be included, based on the contributions from the other frames that were not deleted. Conversely, new localizations can arise by deleting a frame if an upper limit on the localization precision is imposed (e.g. to exclude bright fiduciary markers). In this case, there is a chance that the localization precision becomes sufficiently large upon deleting one of the frames where it was visible. Both problems can be solved by re-estimating the localization precision after the deletion of one frame, as shown in Supplementary Figure 14b. This can be done by making two approximations: (1) the number of photons in each frame is constant, and (2) the localization precision is inversely related to the square root of the amount of photons. An emitter with localization precision σ that appeared in n frames therefore obtains a new localization precision after deleting one frame given by

$$\sigma_{\text{delete-1}} = \sigma\sqrt{n-1} \tag{30}$$

After re-calculating the localization precisions and applying the upper and lower limit on the localization precision, the N new SMLM images for the SNR calculation can be rendered.

To calculate the variance in Eq. 27, a sufficient number of localizations have to be present inside the pixel area. If not the case, for instance due to a too small pixel size or a too low localization density, the SNR value can become unreliable.

3.3 SNR estimation on SOFI data

When calculating SOFI for long image sequences, photobleaching cannot be neglected. The full image sequence is therefore divided into short subsequences during which the photobleaching effect is insignificant. In our case, each subsequence contained 500 frames. For decreasing the computational burden while evaluating the jackknife resampling, the SOFI image is first pre-calculated for each subsequence. The resampling is always performed within one subsequence, then the pre-calculated SOFI images from the remaining subsequences are added to generate a new resampled SOFI image, as shown in Supplementary Figure 15. At the beginning, the algorithm takes the first subsequence (the first 500 frames) from a total number of K subsequences. The first frame from this subsequence is discarded. A SOFI image s_1 is calculated from the rest of the subsequence (i.e. the following 499 frames). The SOFI image s_1 is summed with the pre-calculated SOFI images from the remaining $K-1$ subsequences which yields a resampled SOFI image $I_1(x, y)$. In the next step, the second frame is discarded, leaving a different subset of 499 frames used to calculate a SOFI image s_2. Combining s_2 with the pre-calculated SOFI images from the remaining $K-1$ subsequences yields a resampled SOFI image $I_2(x, y)$. When the whole first subsequence is resampled, the procedure is repeated step by step for every subsequence to cover the full image sequence (i.e. 20,000 frames in our data).

3.4 SNR convergence rate

The SNR increases with an increasing number of frames used for the super-resolution image reconstruction. Supplementary Figure 8 shows the SNR ramp up, i.e. how quickly the SNR converges to a value that remains almost stable at a plateau with almost no further improvement due to an increasing number of frames. The relative increment of the SNR shown in Supplementary Figure 8 was calculated as

$$\mathrm{ISNR} = \frac{|\mathrm{SNR}_{n+1} - \mathrm{SNR}_n|}{|SNR_n|} \tag{31}$$

The measured SNR values are shown in Figure 1.

Supplementary Note 4: Resolution based on MTF analysis

Modulation transfer function (MTF) analysis on simulated data allows us to compare the spatial-temporal resolution of PALM and SOFI under controlled conditions close to the conditions in focal adhesions.

Comparing PALM and SOFI is challenging due to their very different nature (i.e. a list of localizations vs. higher order statistics calculated across the input image stack). Measures like precision, recall or accuracy are often used when comparing PALM algorithms. In this case, a list of localized emitters is compared with the ground truth data. This approach is not well suited for comparing PALM and SOFI. Although the image resolution improves with increasing SOFI order, SOFI does not provide the localizations of underlying emitters.

Therefore, we propose a new approach based on the MTF analysis of a simulated test pattern. We extended the MTF analysis, already well known from classical optics, for application in super-resolution imaging. This generalized MTF analysis uses the same terminology like the visibility and the cut-off frequency for super-resolution and allows one to assess the full path from object to super-resolved image.

4.1 Test pattern

The test pattern is composed of bars with varying width ranging from 500 nm to 20 nm. More precisely, the bars were 500, 400, 300, 200, 150, 120, 100, 90, 80, 70, 60, 50, 40, 30, and 20 nm wide. Repeating every width for three consecutive bars led to the test pattern with 45 bars as shown in Figure 2a. Assuming fluorescent labelling, the bars are filled by uniformly distributed emitters according to a predefined labelling density.

4.2 Simulations and photo-physical model

The simulation assumes photokinetics typical for fluorescent proteins in PALM measurements. For each fluorophore, a time trace is modelled. The time trace describes the number of photons emitted by a given fluorophore over time. Each fluorophore, once it is in the on-state, shows a "burst" of blinking events before being bleached. The blinking fluorophore randomly switches between the on-state and the dark state (Supplementary Figure 13e). On- and off-times of these blinking events, as well as bleaching of the fluorophore, are governed by an exponential distribution with an average on-time τ_{on}, an average off-time τ_{off}, and an average bleaching lifetime τ_{bl}. The on-time ratio $\tau_{\text{on}}/(\tau_{\text{on}} + \tau_{\text{off}})$ defines the frequency of the blinking in the burst. Assuming a camera frame rate of 50 Hz, the blinking parameters were set in order to obtain a similar behavior as mEos2 measured in [2]. The average duration of 8 blinking events in one burst takes on average 3.2 s (on-time ratio = 0.05). The exposure time is assumed to be faster than τ_{on} and τ_{off} and the blinking is therefore properly sampled. Supplementary Figure 13a shows time traces of the first 10 fluorophores. Please note that Supplementary Figure 13a shows the time traces before adding noise. The number of blinks per burst is random (in the range 2-10). Supplementary Figure 13c,d shows the statistics of the simulated image stack. The average number of blinks per burst is equal to 5.9. Supplementary Figure 13c shows the number of photons as a function of frame number normalized to one. An exponential decay was fitted to measure the average bleaching lifetime. Bleaching in the simulation was set to match our experimentally measured data.

4.3 Modulation transfer function (MTF) and sFRC

For our MTF analysis, the pattern consists of progressively narrower black and white bars. When imaging this pattern, the bars might still be resolved, but the visibility decreases with

increasing spatial frequencies. The visibility is given as

$$M = (F_{\max} - F_{\min})/(F_{\max} + F_{\min}) \tag{32}$$

where $F_{\max}$ and $F_{\min}$ are taken as the maximum and minimum intensity values at a given spatial frequency. In classical optics, the microscope is described as a low pass filter. The MTF describes this filtering effect when comparing a periodic object (with a given spatial frequency) to the filtered image. The MTF can be calculated as

$$\mathrm{MTF} = |\mathcal{F}\{P(\mathbf{r})\}| \tag{33}$$

where $P(\mathbf{r})$ represents the test pattern and the operator $\mathcal{F}\{\}$ corresponds to the Fourier transform. The modulation depth is associated to the aforementioned visibility and this generalized MTF analysis integrates all contributions starting from the object and ending with the super-resolved image. Assuming no noise, the cut-off frequency f_c corresponds to the spatial frequency where the visibility goes to zero and the limit of resolution is given by $1/f_c$.

The resolution measured by the MTF analysis (rMTF) can be related to the resolution based on sFRC. The test pattern described in Supplementary Note 4.1 is a rectangular object with spatial frequencies changing along one direction. These conditions are not suitable for the standard FRC calculation. Therefore, we have calculated the sFRC on a segment in the direction which corresponds to the main spectral content in the Fourier space. Supplementary Figure 5a,b shows a comparison of the sFRC and rMTF for a labelling density of 800 and 1200 molecules per μm^2, respectively. Interpretation of the sFRC values should be done carefully, as the spatial resolution value obtained from the MTF analysis is typically slightly lower.

Supplementary Note 5: Correcting blinking events in PALM data

PALM can be used to obtain quantitative molecular information of focal adhesions, such as the number of fluorescent proteins and the blinking off-times. However, simply counting the localizations usually yields an overestimation of this quantity, since fluorescent proteins are known to blink. This error can be avoided by merging localizations that are close in time and space. However, applying such counting methods on focal adhesion data is challenging, since focal adhesions are dense protein structures. We have, therefore, adapted a counting method to take higher localization densities into account.

5.1 Spatial and temporal clustering of blinking events

Counting blinking fluorescent proteins from PALM data can be done as published in [13]. If two different localizations x_{a} and x_{b} are sufficiently close and observed within a sufficiently small time interval, they can be assumed to originate from two blinking events of the same fluorescent protein. First, all localization pairs with a time interval below a certain threshold value t_{d} are considered as potential blinking events. Next, a second selection is made based on the distance between them, i.e. they are considered blinking events if they are closer than a distance threshold. After merging, the localizations are again evaluated against the same criteria until no blinking events can be identified. In order to apply the method to correct for blinking, the value of t_{d} is varied in multiples of the camera exposure time t_{exp}. For each t_{d} the total number of localizations $N(t_{\mathrm{d}})$ is determined, and these values are fitted to the following semi-empirical model in order to obtain the correct number N of fluorescent proteins [13]

$$N(t_{\mathrm{d}}) = N(1 + n_{\mathrm{blink}} e^{\frac{t_{\mathrm{exp}} - t_{\mathrm{d}}}{t_{\mathrm{off}}}}) \tag{34}$$

where n_{blink} is the average number of times a fluorescent protein blinks and t_{off} is the average time between two blinking events. This model assumes that the fluorescent protein first goes from an off-state to an on-state. Once the protein is activated, it either reversibly goes to a dark state or irreversibly to a photobleached state (see Supplementary Figure 13e). For large values of t_{d}, the model predicts that the observed $N(t_{\mathrm{d}})$ approaches N, as would be expected. However, the larger the value of t_{d}, the higher the probability of grouping localizations from different fluorescent proteins, which is not accounted for by the model. Hence, the fit is only performed for small values of t_{d}, i.e. the first 5 multiples of t_{exp}, as suggested in [13].

5.2 Distance threshold accounting for localization precision

A single distance threshold value for all localizations should be avoided, since the localization precision σ_{a} and σ_{b} corresponding to x_{a} and x_{b}, respectively, can be very different. Consider the observed localizations x_{a} and x_{b} to be normally distributed around the real protein positions μ_{a} and μ_{b}, respectively, with standard deviation σ_{a} and σ_{b}, respectively. The question whether both localizations are originating from the same emitter thus boils down to the question how similar both normal distributions are. We therefore defined a threshold based on the Hellinger distance, a statistical measure that probes the similarity between two normal distributions. The Hellinger distance H can be calculated from the following expression

$$H^2 = 1 - \sqrt{\frac{2\sigma_{\mathrm{a}}\sigma_{\mathrm{b}}}{\sigma_{\mathrm{a}}^2 + \sigma_{\mathrm{b}}^2}} exp(-\frac{1}{4}\frac{(\mu_{\mathrm{a}} - \mu_{\mathrm{b}})^2}{\sigma_{\mathrm{a}}^2 + \sigma_{\mathrm{b}}^2}) \tag{35}$$

The Hellinger distance varies between 0 and 1. It is equal to 0 if both probability distributions are identical, and it is equal to 1 if the two probability distributions do not overlap. A threshold value of 0.9 is a reasonable choice, since it corresponds to a distance threshold between two

localizations equal to ~ 3.6 times the localization precision, assuming that their localization precisions are equal. As the real positions are not known, we approximate $\mu_\mathrm{a} - \mu_\mathrm{b}$ by $x_\mathrm{a} - x_\mathrm{b}$ in order to calculate the Hellinger distance.

5.3 Position and localization precision of merged blinking events

The merging procedure is repeated until no blinks can be identified, so one needs to calculate the position and localization precision of merged blinking events. Assuming that the observed localizations are normally distributed around their real positions with the localization precision as the standard deviation, we consider σ_a and σ_b as weights to calculate the new position as follows

$$x_\mathrm{merged} = \frac{x_\mathrm{a}/\sigma_\mathrm{a}^2 + x_\mathrm{b}/\sigma_\mathrm{b}^2}{1/\sigma_\mathrm{a}^2 + 1/\sigma_\mathrm{b}^2} \tag{36}$$

The corresponding localization precision of the merged position is given by

$$\sigma_\mathrm{merged} = \frac{1}{\sqrt{1/\sigma_\mathrm{a}^2 + 1/\sigma_\mathrm{b}^2}} \tag{37}$$

Although the merging procedure was described in one dimension, its application for two dimensional data was done for each dimension separately, i.e. localizations were considered to be blinking events when they were identified as such in both dimensions.

Supplementary References

[1] M. Geissbuehler and T. Lasser, “How to display data by color schemes compatible with red-green color perception deficiencies.,” *Optics express*, vol. 21, no. 8, pp. 9862–74, 2013.

[2] N. Durisic, L. Laparra-Cuervo, A. Sandoval-Álvarez, J. S. Borbely, and M. Lakadamyali, “Single-molecule evaluation of fluorescent protein photoactivation efficiency using an in vivo nanotemplate.,” *Nature methods*, vol. 11, pp. 156–62, 2014.

[3] N. Banterle, K. H. Bui, E. A. Lemke, and M. Beck, “Fourier ring correlation as a resolution criterion for super-resolution microscopy,” *Journal of Structural Biology*, vol. 183, pp. 363–367, 2013.

[4] R. P. J. Nieuwenhuizen, K. a. Lidke, M. Bates, D. L. Puig, D. Grünwald, S. Stallinga, and B. Rieger, “Measuring image resolution in optical nanoscopy.,” *Nature methods*, vol. 10, pp. 557–62, 2013.

[5] N. B. Vicente, J. E. D. Zamboni, J. F. Adur, E. V. Paravani, and V. H. Casco, “Photobleaching correction in fluorescence microscopy images,” *Journal of Physics: Conference Series*, vol. 90, no. 012068, 2007.

[6] S. Geissbuehler, N. L. Bocchio, C. Dellagiacoma, C. Berclaz, M. Leutenegger, and T. Lasser, “Mapping molecular statistics with balanced super-resolution optical fluctuation imaging (bSOFI),” *Optical Nanoscopy*, vol. 1, no. 1, 2012.

[7] T. Dertinger and R. Colyer, “Fast, background-free, 3D super-resolution optical fluctuation imaging (SOFI),” *Proceedings of the ...*, vol. 106, no. 52, 2009.

[8] T. Dertinger, R. Colyer, R. Vogel, J. Enderlein, and S. Weiss, “Achieving increased resolution and more pixels with Superresolution Optical Fluctuation Imaging (SOFI).,” *Optics express*, vol. 18, pp. 18875–85, Aug. 2010.

[9] S. C. Stein, A. Huss, D. Hähnel, I. Gregor, and J. Enderlein, “Fourier interpolation stochastic optical fluctuation imaging.,” *Optics express*, vol. 23, pp. 16154–63, 2015.

[10] W. Vandenberg, M. Leutenegger, T. Lasser, J. Hofkens, and P. Dedecker, “Diffraction-unlimited imaging: from pretty pictures to hard numbers,” *Cell and Tissue Research*, vol. 360, no. 1, pp. 151–178, 2015.

[11] S. Geissbuehler, A. Sharipov, A. Godinat, N. L. Bocchio, P. a. Sandoz, A. Huss, N. a. Jensen, S. Jakobs, J. Enderlein, F. Gisou van der Goot, E. a. Dubikovskaya, T. Lasser, and M. Leutenegger, “Live-cell multiplane three-dimensional super-resolution optical fluctuation imaging,” *Nature Communications*, vol. 5, Dec. 2014.

[12] W. Vandenberg, S. Duwé, M. Leutenegger, B. Krajnik, T. Lasser, and P. Dedecker, “Model-free uncertainty estimation in Stochastical Optical Fluctuation Imaging (SOFI) leads to a doubled temporal resolution,” vol. 2402, pp. 1347–1355, 2015.

[13] P. Annibale, S. Vanni, M. Scarselli, U. Rothlisberger, and A. Radenovic, “Quantitative Photo Activated Localization Microscopy: Unraveling the effects of photoblinking,” *PLoS ONE*, vol. 6, no. 7, 2011.
